# Nursing Degree Curriculum: Differences and Similarities Between 15 European Countries

**DOI:** 10.3390/nursrep15030112

**Published:** 2025-03-19

**Authors:** Celeste Antão, Bruna Santos, Nelson Santos, Hélder Fernandes, Bárbara Barroso, Cristina Oana Mǎrginean, Helena Pimentel

**Affiliations:** 1 Research Centre for Active Living and Wellbeing (LiveWell), Instituto Politécnico de Bragança, Campus de Santa Apolónia, 5300-253 Bragança, Portugal; celeste@ipb.pt (C.A.); hpimentel@ipb.pt (H.P.); 2 Instituto Politécnico de Bragança, Campus Santa Apolónia, 5300-253 Bragança, Portugala51245@alunos.ipb.pt (N.S.); 3 CeDRI—Research Center in Digitalization and Intelligent Robotics, Instituto Politécnico de Bragança, Campus de Santa Apolónia, 5300-253 Bragança, Portugal; bbarroso@ipb.pt; 4 Department of Pediatrics, “George Emil Palade” University of Medicine, Pharmacy, Science and Technology of Târgu Mureş, 540142 Târgu Mureş, Romania; marginean.oana@gmail.com

**Keywords:** nursing, curriculum, Europe

## Abstract

This study explores the curriculum of Nursing Bachelor’s degrees across 15 European countries, aiming to describe nursing course curricula and admission requirements and to identify differences and similarities in the implementation of the Bologna Process. The research employs a qualitative and documentary methodology, analyzing curricula from selected higher education institutions involved in the Erasmus+ “Innovative Skills for Nurses” project. The findings reveal variations in the duration of training, with some countries adopting 3-year (180 ECTS) programs, while others require 4-year (240 ECTS) programs. Furthermore, discrepancies were found in the balance between theoretical and clinical education, as well as the availability of optional subjects. Countries with longer training programs tend to offer more consolidated practices and greater alignment with Bologna’s principles, fostering better professional outcomes. The study highlights the challenges posed by non-standardized training durations and their impact on mobility and employability of nursing professionals. These findings may inform future discussions on harmonizing nursing education across Europe to ensure consistency in quality and professional competencies.

## 1. Introduction

The demanding context of change we are experiencing emphasizes the need for training systems that promote inclusion, sustainability, resilience, and adaptability. This aims to enable professionals, both theoretically and practically, so they are equipped to keep pace with changes and expand their areas of expertise. In nursing, this need assumes a prominent role as the foundation for excellent professional practice, which acts as a differentiating and determining factor in terms of employability, adaptability, professional development, and personal fulfillment [1].

On 19 June 1999, 29 countries signed the Bologna Declaration with the intention of standardizing higher education systems across Europe. This declaration encourages the mobility of students, teachers, and researchers, ensuring high-quality education while making it more accessible and inclusive. To place students at the center of the process, a new system of curriculum credits (ECTS)—European Credit Transfer System—was created, replacing the previous system. This system became one of the most important tools of this European policy, focusing on both the organization of curriculum units and the evaluation and crediting, now considering the entirety of the work involved in the student’s education process [2].

In addition, the Bologna Process introduced a three-cycle education system, corresponding to bachelor’s, master’s, and doctoral degrees [3,4,5]. In structuring these three cycles according to competencies, knowledge, and skills, the Dublin Descriptors are used. These are organized into five categories: Knowledge and Understanding; Application of Knowledge and Understanding; Judgment Formation; Communication Skills; and Learning Skills. In the case of a bachelor’s degree, the Dublin Descriptors assume that students will be able to grasp and apply knowledge, understand and solve problems in a specific field of study. It is also expected that they will be able to gather, select, and interpret information in their area of specialization to support decisions based on social, scientific, and ethical aspects. Furthermore, the Dublin Descriptors assume that the learning process should be continuous throughout life, allowing for a high degree of autonomy and updating. The descriptors aim for students to be capable of communicating information, ideas, problems, and solutions to both specialized and non-specialized audiences [6].

The curriculum of a Nursing Bachelor’s degree program includes two components: a theoretical component and a clinical education component, which are interconnected. The theoretical component aims to equip students with scientific, ethical, moral, and professional knowledge, enabling them to justify their actions in practical contexts. This component should focus on the teaching of Nursing Care, Fundamental Sciences, and Social Sciences. Clinical education serves as a means for acquiring important knowledge and skills for nursing practice. This is provided through supervised internships by qualified teachers and professionals in health units or within the community. In this context, the training covers topics related to General Medicine and Medical Specialties, General Surgery and Surgical Specialties, Pediatric Care and Pediatrics, Hygiene and Maternal and Newborn Care, Mental Health and Psychiatry, Elderly Care and Geriatrics, and Home Care [7,8].

For the first cycle of studies, the Bologna Process proposes a 180 ECTS training program, corresponding to three academic years. However, some professions require longer training periods, particularly in health-related fields such as nursing education [2]. Directive 2005/36/EC of the European Parliament and of the Council of 7 September 2005 on the recognition of professional qualifications governs the mutual recognition of qualifications in regulated professions. Nursing training seeks to respond to new challenges, balancing the richness of tradition with the need to address new perspectives and greater responsibilities.

The move to higher education and the adaptation of nursing education to the Bologna Process is nothing more than the recognition that today’s nurses need a broad education to help them successfully face the demands of a changing society.

According to the same source, one of the ‘novelties’ of the Bologna Process was the universalization of curricular credits, expressing both the work that must be done by the student in each curricular unit and the scientific area in which it is integrated. Additionally, greater practical contact, whether through practical classes or clinical teaching/internships, enhances the ability to solve problems that may arise in a real-life context.

Higher education institutions have the right to create cycles of studies aimed at conferring academic degrees. The establishment of such study programs requires accreditation by the Agency for Assessment and Accreditation for Quality Assurance in Higher Education and subsequent registration with the relevant ministry.

Higher education institutions may freely establish association or cooperation agreements among themselves or with other institutions to encourage student and teaching staff mobility and to pursue joint partnerships and projects, including joint degree programs, under the terms of the law. They may also share resources or equipment, either on the basis of territorial aggregation criteria or sectoral aggregation criteria.

Public higher education institutions enjoy statutory, pedagogical, scientific, cultural, administrative, financial, patrimonial, and disciplinary autonomy from the state, with differentiation appropriate to their nature. Each higher education institution has its statutes, which, in compliance with the law, set out its mission, pedagogical and scientific objectives, autonomy, and organizational structure [9].

Similarly, increased practical experience, either through practical classes or clinical education/internships, enhances problem-solving capacity in real-life contexts. Although there has been a European directive on health education since 2005 [1], in the transition to the Bologna model, nursing education did not undergo a uniform standardization, neither in terms of duration nor the number of ECTS. This diversity in training has not contributed to the profession’s affirmation and consolidation [4]. Additionally, student mobility in Erasmus programs and the later mobility of nursing professionals have proven to be more difficult.

As part of the “Innovative Skills for Nurses” project of the Erasmus+ Program, this research aims to analyze the curriculum of fifteen European countries. Therefore, the aim of this study is to analyze the curriculum and entry requirements for nursing courses and to identify differences and similarities in the implementation of the Bologna Process (this includes Poland, Austria, Romania, Portugal, Italy, Spain, Greece, Croatia, the Netherlands, France, Belgium, Germany, Finland, Lithuania, and Estonia). Thus, this project can have an enlightening and reflective impact by exposing the main differences and similarities in curriculum development and teaching methodologies.

## 2. Materials and Methods

To address the research question, “What is the curriculum structure, entry conditions, and the main differences in the Nursing Bachelor’s degree in 15 European countries”? we employed a qualitative methodology. Through this methodology, we aimed to provide new perspectives on nursing education [10]. This study is documentary in nature, as it is based on the description of the reality in the 15 countries involved in this project. Since the situation was described at a single point in time, we conclude that this study is also cross-sectional in nature [11]. The methodology of this study is also classified as documentary, as we relied on foundational documents to find complementary information, enabling the progression of the study group [12].

We considered the curricula of three higher education institutions from each country mentioned earlier as part of the “Innovative Skills for Nurses” project, within the Erasmus+ program. Data were collected by analyzing a report published under this project [13].

Each partner in the “Innovative Skills for Nurses” project (Romania, Portugal, Greece, Lithuania, and France) researched nursing curricula in three countries: their own and two other geographically close countries.

Romania, as project coordinator, compiled all the information and wrote the report. Data were obtained from the websites of nursing education institutions, official websites, and legislation. Each project partner researched three educational institutions in each country.

## 3. Results

Based on the analysis of Table 1, we can observe that the basic nursing education (bachelor’s degree) lasts four years in seven countries and three years in the remaining six countries (240 and 180 ECTS, respectively). Two countries have a 3.5-year program with 210 ECTS. Regarding the number of training hours, the table shows that in most cases, the number of theoretical hours is lower than the number of practical hours/clinical education, except in Croatia, Greece, and France. Concerning the number of hours of clinical education/internships, Portugal stands out for having a high number of hours.

Table 2 outlines the various admission requirements for Nursing Bachelor’s degree programs. Countries such as Germany, France, the Netherlands, Croatia, and Estonia require a secondary school diploma. In Belgium, candidates who do not possess a secondary school leaving certificate can take an entrance exam called the “Paramedical Jury A1”, organized by the French Community of Belgium.

In addition to a secondary diploma, Belgium, Spain, and Croatia also require candidates to pass a specific entrance exam. In contrast, Italy and Portugal mandate both a national biology and chemistry exam and a secondary school diploma.

Language proficiency is a requirement in Germany, Austria, Belgium, and Finland. Additionally, letters of recommendation and motivation are necessary for admission in Poland, Austria, Romania, and Finland.

The description of each country’s curriculum strengths (Table 3) reveals that half of the countries offer 240 ECTS programs, which include a significant clinical practice component. Most programs emphasize the development of interdisciplinary and interprofessional skills and competencies, and there is a wide variety of internship opportunities.

In Lithuania, practical training is conducted in laboratories and simulation centers. In the Netherlands, nursing education is delivered in a safe, high-tech environment, with a strong focus on public health, innovation, and the deepening of clinical reasoning and practice.

Regarding weaknesses, Austria, France, Italy, Belgium, Croatia, and Poland only offer 180 ECTS programs. Austria’s curriculum does not specify the number of practical hours and places greater emphasis on theoretical training. Greece does not promote research, while Lithuania and Estonia face challenges due to weak social and digital competencies among students.

In France, insufficient development in quality and risk management approaches undermines preparation for inherently high-risk practices. Additionally, complementary therapies and proficiency in other European languages are generally lacking across most countries studied.

Table 4 indicates that in most countries, nursing training is conducted exclusively in the native language. However, in the Netherlands, Italy, Romania, and Finland, courses are also offered in an additional language, allowing for instruction in two languages.

Optional curricular units are available in Portugal, Spain, and Romania. Information on the availability of optional units in other countries was not provided.

## 4. Discussion

Regarding curriculum structure of 15 counties, there are similarities, differences, strong points, weaknesses, number of years, semesters, number of hours, and number of ECTS.

The duration of theoretical education should account for at least one-third of the total workload, while clinical education should last at least half of the total hours [1,7]. In complying with this directive, Portugal has chosen to divide theoretical education and clinical education/internships equally, allocating 120 ECTS to each component [14]. Furthermore, the subjects that comprise nursing education can be annual or semester-based. There is also the possibility for students to enroll in optional theoretical or practical subjects, which are provided based on the capacity and availability of each educational institution, not exceeding 10% of the total workload of the curriculum [14].

Considering the curricula analyzed, only half of the countries included in this study have a longer training plan. In these countries, the first cycle of nursing education lasts four years with 240 ECTS, which, in our view and supported by legislation, leads to a more consolidated education. In countries where this education lasts three years and consists of 180 ECTS, there is necessarily an overload of contact hours in classroom settings, leaving little or no time for independent work, which goes against the principles advocated by the Bologna Process. Furthermore, a lower number of hours dedicated to practical clinical education and/or internships leads to less secure and consolidated practical experiences, making it more difficult to assert professional recognition and employability, as well as hindering mobility. Despite this diversity, we found that in all 15 countries, the same scientific areas are covered in nursing education, albeit in different ways. An example of this can be seen in the subject of Anatomy. In Portugal, this subject is taught independently and is generally called Anatomohistophysiology, while in France, this same subject is distributed across different curriculum units [15,16,17].

We also see significant advantages and competitiveness in some countries offering education in more than one language. The globalization of education, whether through student mobility or the option to study in a different country from one’s own, is undoubtedly an added value that this possibility provides.

Higher education, in addition to providing knowledge specific to a particular area, should also enrich multidisciplinary experiences. This is achieved through optional curriculum units [18]. However, not all countries offer this possibility in their curricula, as is the case with Poland. Spain, for example, offers third-year students the opportunity to choose theoretical subjects, while in Portugal, third-year students are allowed to choose optional internships.

According to Nunes, during the analysis phase of adapting nursing education to the Bologna Process, significant events supported the defense of a unified nursing education system in Europe. In June 2000, the second WHO Ministerial Conference on Nursing and Midwifery in Europe was held with the aim of establishing the fundamental principles for the initial training of nurses and midwives [19].

In 2005, a report by the World Health Organization—European Region highlighted the need to regularly evaluate progress and use the results politically to improve the state of nursing and midwifery education across Europe [20]. In 2009, the “Global Standards for the Initial Education of Professional Nurses and Midwives” were published [21].

Following Bulgaria and Romania’s accession to the European Union, Law No. 9/4 March 2009 [22] transposed into national law Directive 2005/36/EC of the European Parliament and Council of September 7, concerning the recognition of professional qualifications, and Directive 2006/100/EC of the Council of November 20 [23], which adapted certain directives on the free movement of people. This established the applicable regime in Portugal for recognizing professional qualifications obtained in another EU member state by nationals of an EU member state who wish to practice a regulated profession, either as an independent or subordinate worker, that is not covered by another specific regime.

Several European Federations in this field aligned with the expectation of increasing the competitiveness of European nursing and promoting the roles and influence of nurses on the health status of individuals, families, and communities in Europe. This alignment aimed to make European higher education in nursing more comprehensible and attractive to students, academics, and employers both within the EU and globally [19]. Furthermore, according to the same author, in 2004, the FEP was established, later becoming the European Nurses Council (ENC), as an association of nursing regulators. The European Federation of Nurses Associations (EFN) had already existed since the 1970s, and the countries that created the FEPI were EFN members but sought to focus their lobbying efforts more on self-regulation. Additionally, the European Federation of Nursing Educators (FINE) was established in the 1990s.

Ordinance No. 799-D/99 provides for the possibility of this training offering students 10 ECTS of optional curricular units. Portugal complies with the stipulated requirements for both theoretical and practical components, providing students with the opportunity to pursue interests in areas of study that, while not necessarily directly related to nursing, contribute to their holistic development [7]. This also facilitates the realization of Erasmus mobility opportunities among the student population [4].

With regard to the conditions for admission to nursing courses, all programs require a secondary school diploma. However, the number of years required for this education is not always explicitly stated. Belgium, Spain, and Croatia require an entrance exam, while Italy and Portugal require specific exams in biology and chemistry.

Proficiency in the native language (both oral and written) is mandatory in almost all countries (Germany, Austria, Belgium, and Finland). This is an important prerequisite for the development of communication skills with the user/family and multidisciplinary team, as well as the ability to critically and reflexively analyze the learning context and respond appropriately to the individualization of care.

Admission requirements vary widely, ranging from national university entrance exams to specific national tests in biology and chemistry. Some countries also require letters of motivation and recommendation.

The curricula in Portugal and Poland include a significant proportion of practical hours (55.93%), similar to the curricula in Romania. Additionally, the curricula in Romania, Portugal, the Netherlands, Greece, Spain, and Germany span four years, comprising eight semesters and 240 ECTS, in compliance with EU directives [1,3,12].

Each country has developed a sufficiently comprehensive curriculum in terms of skills and knowledge, starting with theoretical foundations and incorporating a range of transversal curricular units before progressing to more specialized nursing training. In some countries, training emphasizes innovation, research, and simulated practice as key strengths. The practical component, developed within clinical contexts, enables students to acquire interpersonal, instrumental, and systematic skills. Longer curricula (four years/240 ECTS) provide opportunities for clinical learning across a broad spectrum of specific areas, from maternal and child care to geriatric and palliative care. In contrast, shorter curricula appear to compromise this breadth of clinical learning. This observation aligns with Directive 205/36/EU, consolidated in Directive 2013/55/EU [1,24].

Similarly to previous findings [25,26], our results indicate that despite different educational solutions and curricula, based on their self-assessments, Graduating Nursing Students (GNSs) are graduating with a good level of competence, but competence levels still differ across EU countries. According to the same authors, since 2015, about 67,000 nurses with professional qualifications obtained in one EU country have applied for recognition of their qualifications in another country for permanent practice. This reality highlights the relevance of graduated standardization in nursing, as advocated by EU directives [1,2].

However, as our results show, there are still differences, particularly in the length of graduate education and the amount of time spent learning in a clinical setting, which are reflected in the skills needed to practice the profession. The need to acquire highly qualified nursing skills has already been highlighted in a broader analysis of 29 European countries [27].

Among the weaknesses identified across countries are shorter training durations (three years/180 ECTS), which particularly impact clinical training. Additionally, most programs are delivered exclusively in the native language. This reality reflects a limitation to the mobility of students, nurses, and researchers already mentioned by other studies [26,27]. The second authors also recommend that Member States publish at least in English and later, if possible, in all official EU languages.

There is a limited range of optional curricular units. These variations result in diverse levels of knowledge and competencies within the European nursing education framework.

This disparity explains why mobilizing and employing professionals from countries with longer nursing training is easier. Furthermore, this reality impacts the admission criteria and the design of postgraduate programs aimed at acquiring advanced and specialized nursing skills.

A limitation of this study is the difficulty in standardizing the information in the nursing education plans of the 15 countries described. Only three plans from each country provided sufficient data for detailed description. With the adaptation to Bologna, schools and faculties have increased their scientific and pedagogical autonomy and may have different curricular structures within the same country.

## 5. Conclusions

The adaptation of higher education to the Bologna Process aimed to standardize first-cycle education with 180 ECTS, corresponding to three years of training. However, some professions, especially those in the healthcare sector, like nursing, considering European legislation for the practice of these professions, implemented longer training periods—a fact observed in half of the countries analyzed within the scope of the “Innovative Skills for Nurses” project.

Extended training fosters more stable and consolidated practices and a greater capacity to respond to real-life situations, as well as to increasingly global healthcare challenges. The countries that maintain a three-year education system still face an overload of contact hours and a reduced amount of time for independent work, which is misaligned with the principles that the Bologna Process sought to implement.

In the training plans of the 15 countries studied, all scientific areas are addressed, though in different ways, as exemplified with the subject of Anatomy. Legislation recommends that the curriculum structure should include at least one-third of theoretical hours and that half of the total hours should be dedicated to clinical education/internships. However, this is not always reflected in all the curricula analyzed. Moreover, in most of the curricula, there is the possibility of enrolling in optional subjects, which can account for up to 10% of the total workload.

## Figures and Tables

**Table 1:** Distribution of the number of years of education, ECTS, and number of hours in the curricula of each country.

Countries	Graduation Years	Graduation ECTS	Postgraduation Year	Postgraduation ECTS
Germany	4	240	Included 120 ECTS for the nurses that wished to specialize	
Austria	3	180	*	*
Belgium	3	180	1.0	60
Croatia	3	180	1.0	60
Spain	4	240	*	
France	3	180	2.0	120
Greece	4	240	*	
Netherlands	4	240	1.0	60
Italy	3	180	*	
Poland	3	180	2.0	137
Portugal	4	240	1.5	90 ^1^
Romania	4	240		*
Lithuania	4	240	1.5	90
Estonia	3.5	210	1.5	90
Finland	3.5	210	2.0	120

Source: Own elaboration. ^1^ Two years and 120 ECTS for postgraduation at midwifery education; * The source does not indicate this information.

**Table 2:** Conditions for admission to Nursing Bachelor’s degree in each country.

Countries	Admission
Germany	- 10 years of secondary school education + two years of professional education. - Proof of German language proficiency at B1 level. - University entry exam.
Austria	- Motivation letter reflecting the candidate’s academic and social potential. - Proficiency in German at B2 level (Common European Framework of Reference for Languages).
Belgium	- Candidates must hold the CESS (secondary education diploma) or an equivalent recognized qualification (Baccalaureate). - Candidates without a secondary school leaving certificate can take the “Paramedical Jury A1” entrance exam organized by the French Community of Belgium.
Croatia	- Admission based on achievements in previous education (grades) and results of the State Matura exams.
Spain	- University entrance exam.
France	- Admission to one of the 350 Nursing Training Institutes (IFSI) through a competitive examination. - Requires a baccalaureate diploma; graduates receive a State diploma (Bac + 3) after three years of training.
Greece	- National entrance exam.
Netherlands	- Secondary education diploma.
Italy	- Admission exam conducted entirely in English. - Written test includes 60 multiple-choice questions: 12 General Knowledge, 10 Logical Reasoning, 18 Biology, 12 Chemistry, and 8 Physics and Mathematics.
Poland	- Motivation letter reflecting the candidate’s academic and social potential. - English language proficiency certificate.
Portugal	- Entrance exam from one of the following sets: - Biology and Geology - Biology and Geology & Physics and Chemistry - Biology and Geology & Mathematics
Romania	- Motivation letter reflecting the candidate’s academic and social potential. - Admission test comprising 50 multiple-choice questions on Biology.
Lithuania	- Minimum competitive score: 5.4. - Must pass three state matriculation exams: Lithuanian language and literature, mathematics, and one elective exam. - Average of the five best annual grades (A and B levels) must be at least 7.
Estonia	- Certificate of secondary or vocational secondary education or an equivalent qualification.
Finland	- Proof of language proficiency. - Entrance exam or interview (in some schools). - Health prerequisites and a statement of purpose/letter of intent. - Some universities may require letters of motivation and recommendation.

Source: Own elaboration based in https://innoskillsnurses.umfst.ro/files/WP2/three%20countries%20report/Transnational%20Report.pdf (accessed on 18 March 2025).

**Table 3:** The strengths and weaknesses of main learning outcomes by country.

Countries	Strong Points	Weaknesses
Germany	- Interdisciplinary collaboration with other professional groups. - Multidisciplinary and interprofessional solutions to health problems. - Comprehensive theoretical program and a minimum of 1800 h of practical training. - Employer funding possibilities.	- Program is only offered in German. - Limited focus on anesthesia within the curriculum.
Austria	- Broad spectrum of competencies and highly relevant disciplines such as Nursing Care, Nursing Models and Theories, Psychology, Nursing Diagnostics, and Nursing in Diverse Settings. - Focus on ethics, case management, community care, and quality management. - Strong communication and teamwork skills.	- No specified number of practical training hours. - Only 180 ECTS, with more emphasis on theoretical training than practical skills.
Belgium	- Use of case-based teaching methods. - Specialized professional titles available through short one-year programs.	- Program is only available in French. - Limited clinical internship opportunities. - Lack of European equivalence.
Croatia	- Emphasis on lifelong learning and teamwork. - Prepares students to work with diverse cultural and ethnic communities. - Promotes nursing research and collaboration across medical specialties.	- Insufficient training in practical skills. - Limited promotion of continuous lifelong self-training and digital education (e.g., virtual clinical cases).
Spain	- Focus on autonomous learning and understanding social and multicultural contexts. - Effective communication with patients, families, and colleagues. - Encourages interdisciplinary teamwork and health education.	- Lack of specific knowledge areas in the curriculum.
France	- Selective entry ensures high-quality candidates. - Individualized pedagogical follow-up and support. - Diverse internship opportunities with possible employer financing. - Prepares autonomous and reflective practitioners.	- Limited opportunities for foreign students or French students with foreign qualifications. - Insufficient schools to meet demand. - Training methods are difficult to adapt to other countries. - Limited focus on quality and risk management for high-risk practices.
Greece	- Holistic nursing approach across medical fields. - Strong cooperation with therapeutic teams. - Focus on health promotion and participation in health policy implementation.	- Lack of emphasis on lifelong learning and digital training tools (e.g., virtual clinical cases).
Netherlands	- Emphasis on innovation in healthcare, entrepreneurship, and sustainability. - Use of advanced technologies like 3D printing and simulation training. - High-tech learning environment with a focus on public health.	- Curriculum lacks detailed learning objectives in some areas.
Italy	- Focus on intra- and inter-professional teamwork. - Evidence-based practice and research integration. - Lifelong learning approach supported by strong foundational training.	- Limited to 180 ECTS. - Only 60 ECTS allocated to internships in some universities.
Poland	- Strong theoretical foundation and holistic nursing approach. - Skills include developing therapeutic nursing care plans. - Emphasis on prevention and health education activities.	- Limited to 180 ECTS.
Portugal	- Strong skills in planning, prescribing, executing, and evaluating nursing care. - Focus on research and implementing appropriate intervention strategies.	- Lack of complementary therapies in the curriculum. - Limited inclusion of other European languages.
Romania	- Courses tailored to clinical specialties and subjects like medical sociology, behavioral sciences, and medical ethics.	- Practical activities do not cover a broad enough range of specialties.
Lithuania	- Comprehensive nursing science knowledge. - Practical training in laboratories and simulation centers.	- Weak development of social and digital competencies.
Estonia	- Strong professional skills and e-health system usage. - Practical training in simulation environments before real-world practice.	- Weak development of social and digital competencies.
Finland	- Integration of research and evidence-based nursing with hands-on professional skills. - Focus on technological advancements and practical training. - Emphasis on critical thinking and relevant research in health and social care.	- Language and communication barriers for foreign nursing students.

Source: Own elaboration based on https://innoskillsnurses.umfst.ro/files/WP2/three%20countries%20report/Transnational%20Report.pdf (accessed on 18 March 2025).

**Table 4:** Language used in course instruction in different countries and types of optional curricular units.

Countries	Language	Optional Curricular Units	
		Yes	Not
Germany	German		*
Austria	German		*
Belgium	French		*
Croatia	*		*
Spain	Spanish	Theoretical and practices	
France	French		*
Greece	*		*
Netherlands	Dutch and English		*
Italy	Italian and English		*
Poland	Polish		*
Portugal	Portuguese	Theoretical and practices	
Romania	Romanian and Hungarian	Theoretical	
Lithuania	Lithuanian		*
Estonia	Estonian and English		*
Finland	Finnish and English		*

Source: Own elaboration based on https://innoskillsnurses.umfst.ro/files/WP2/three%20countries%20report/Transnational%20Report.pdf (accessed on 18 March 2025). * The source does not specify data.

## Data Availability

Not applicable.

## Public Involvement Statement

There was no public involvement in any aspect of this research.
